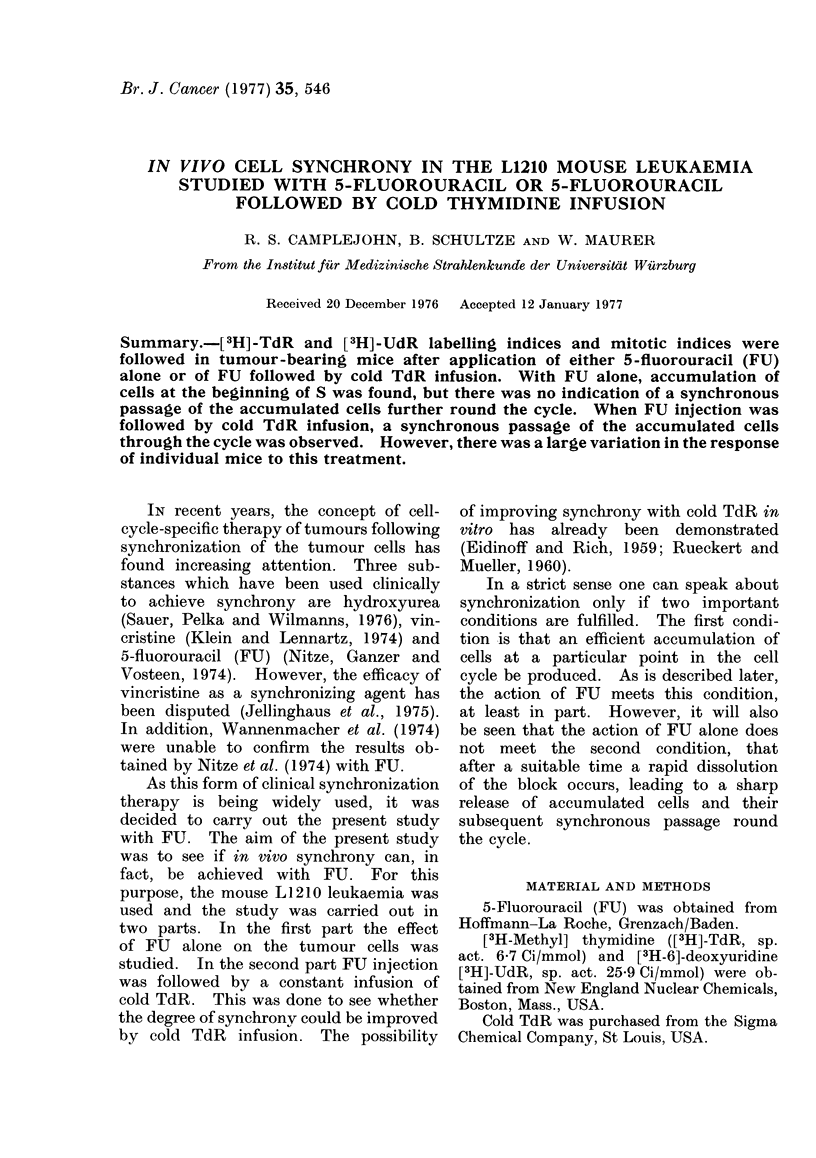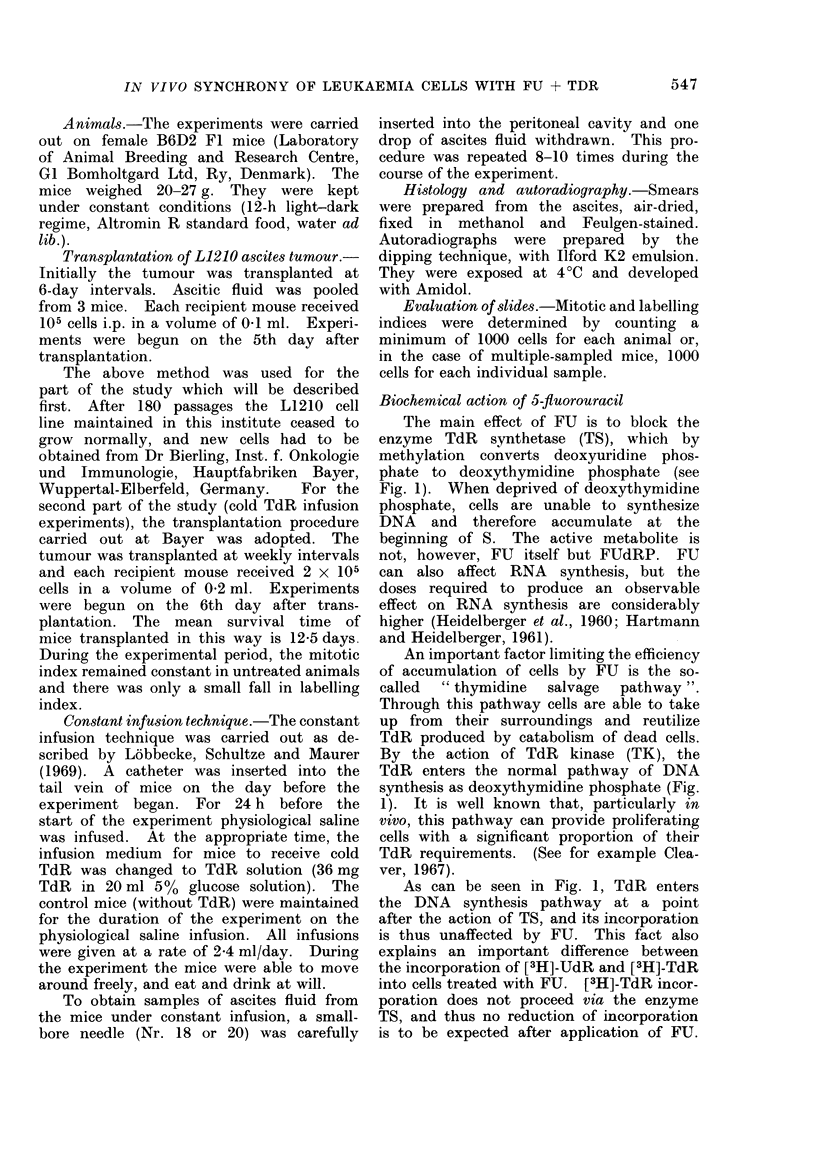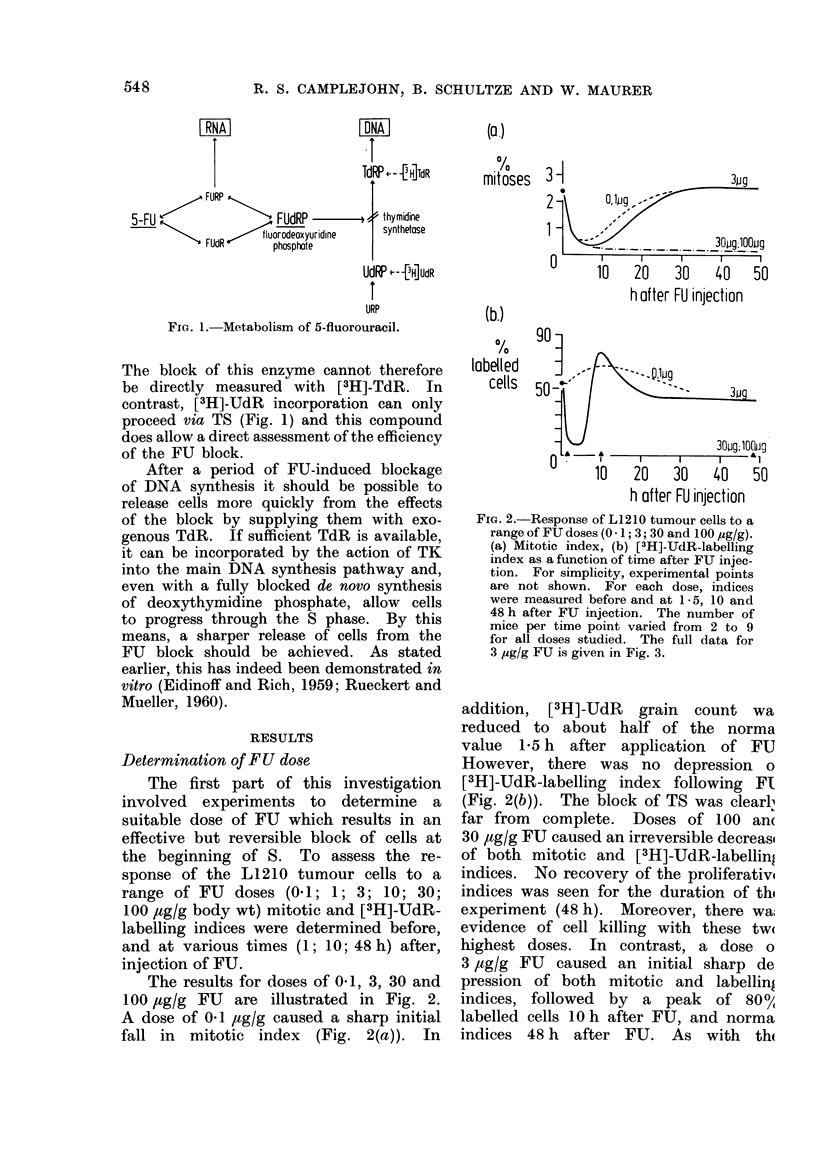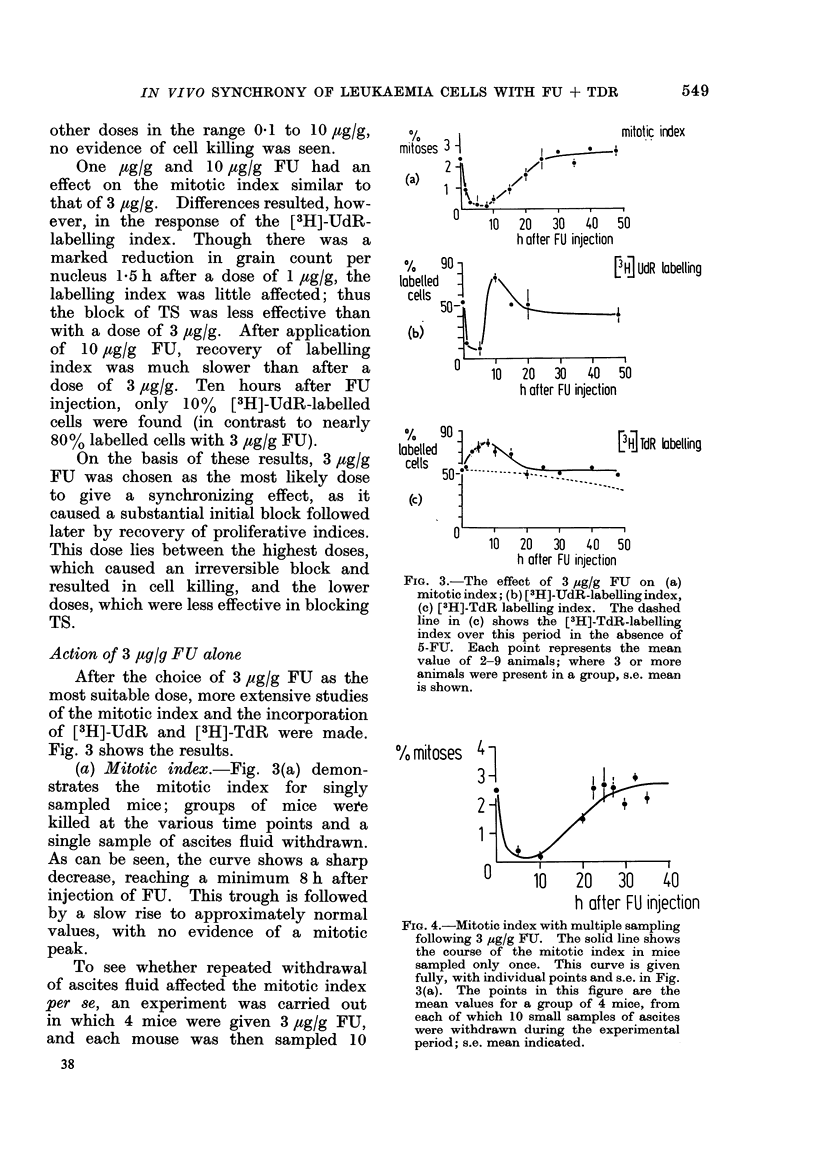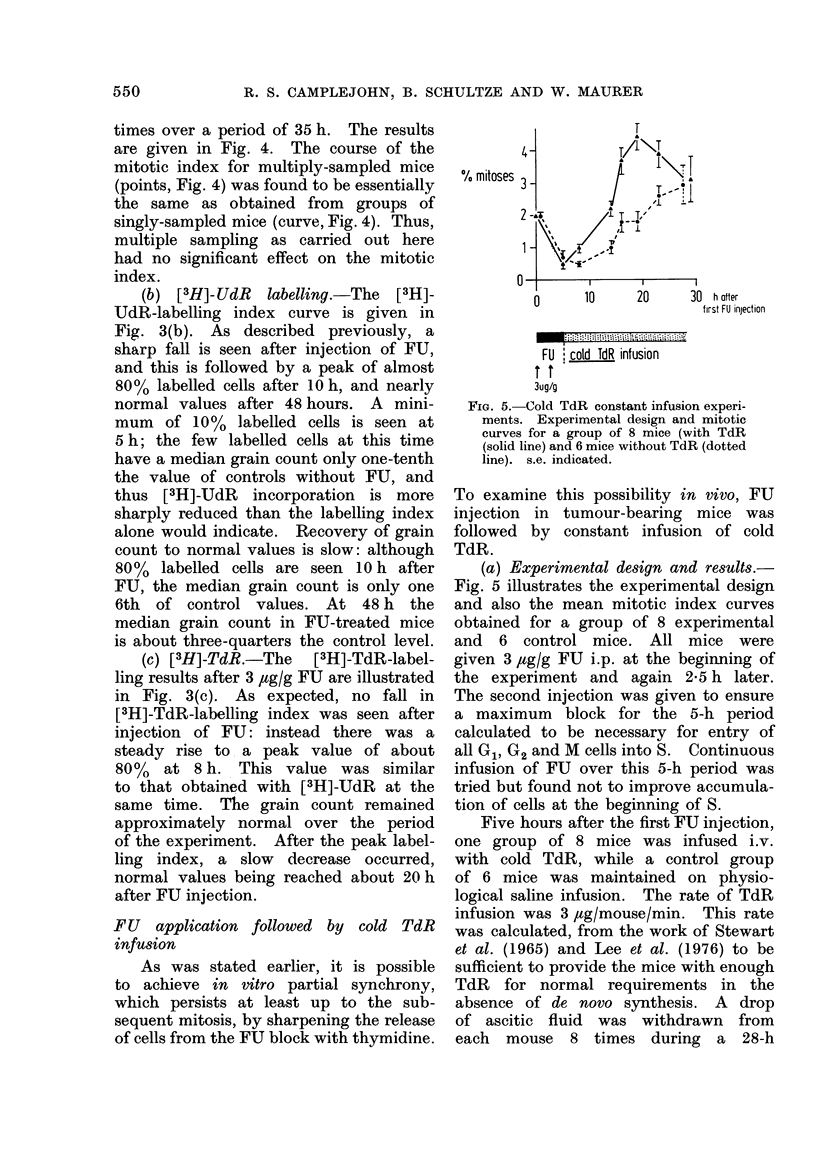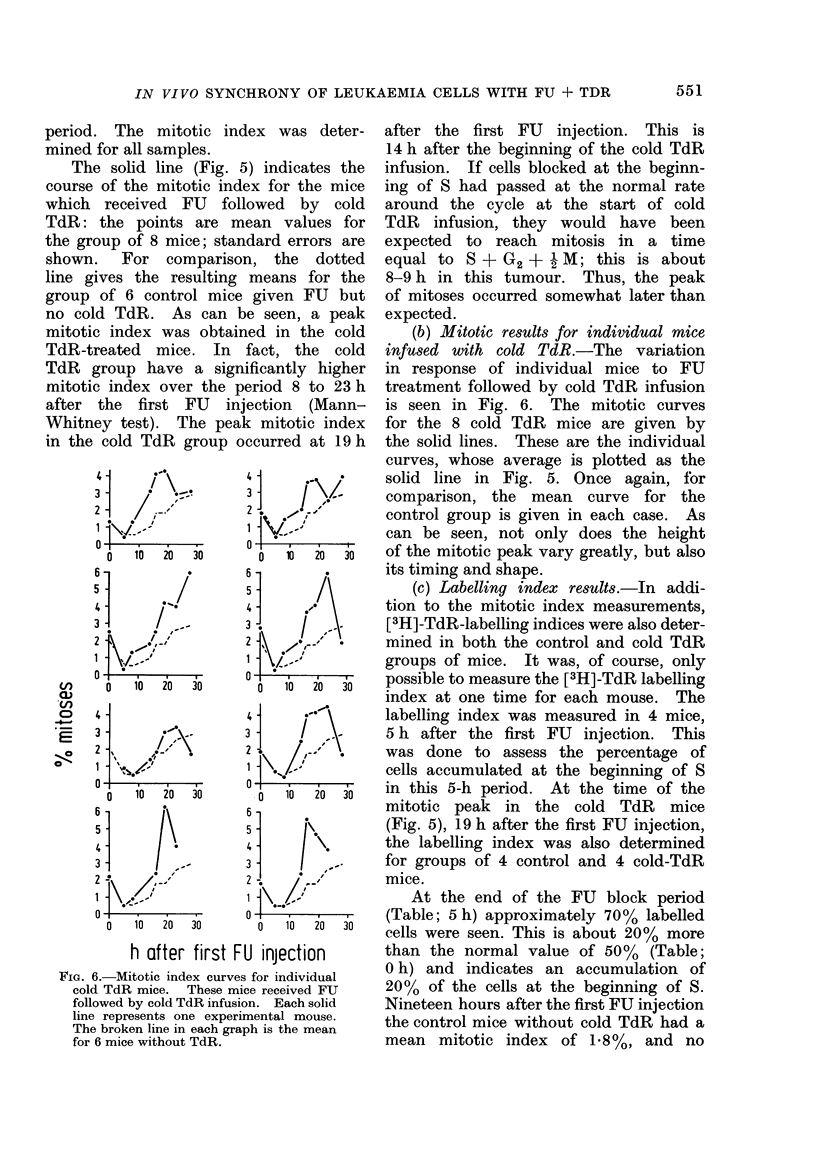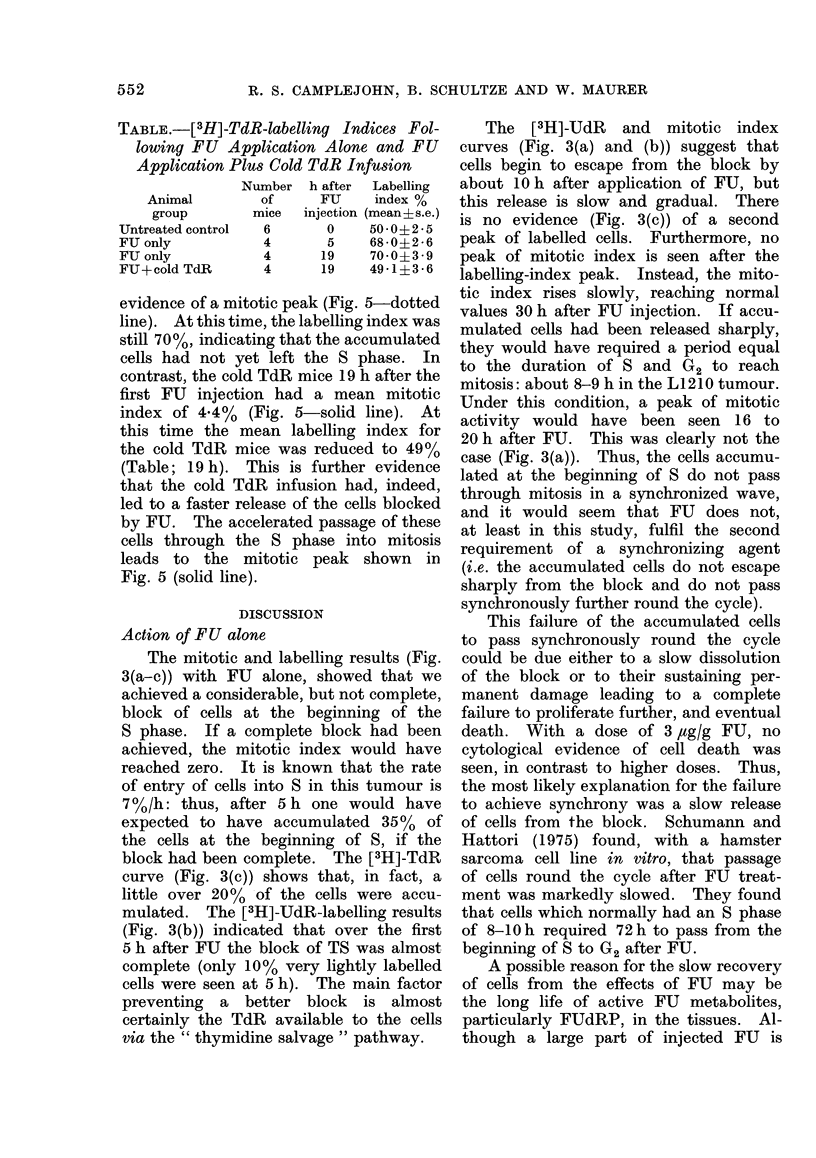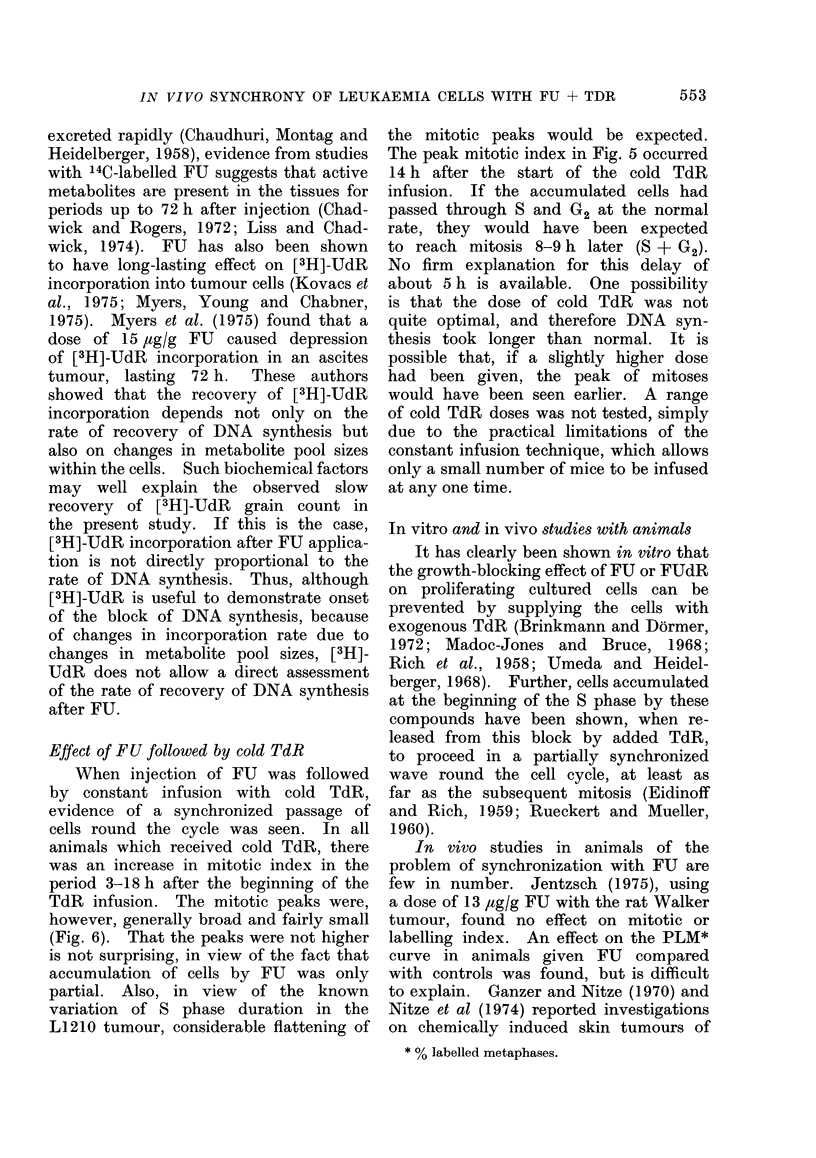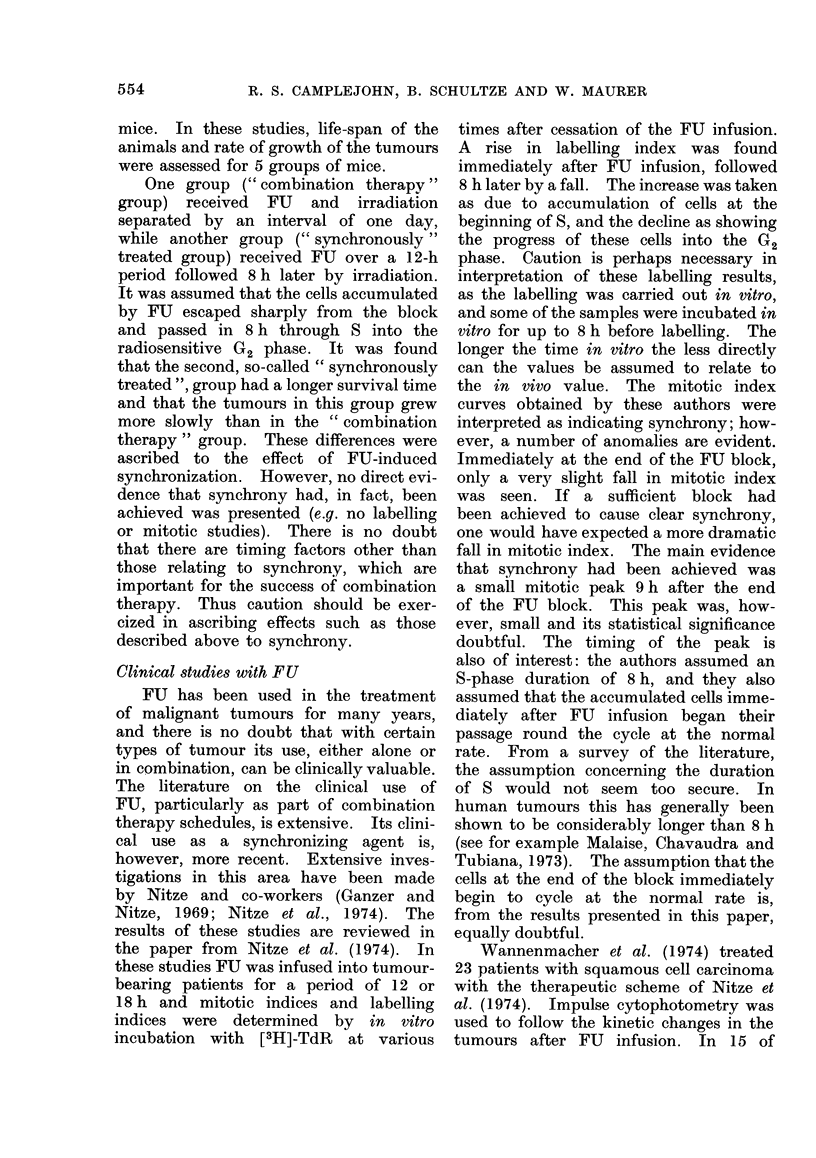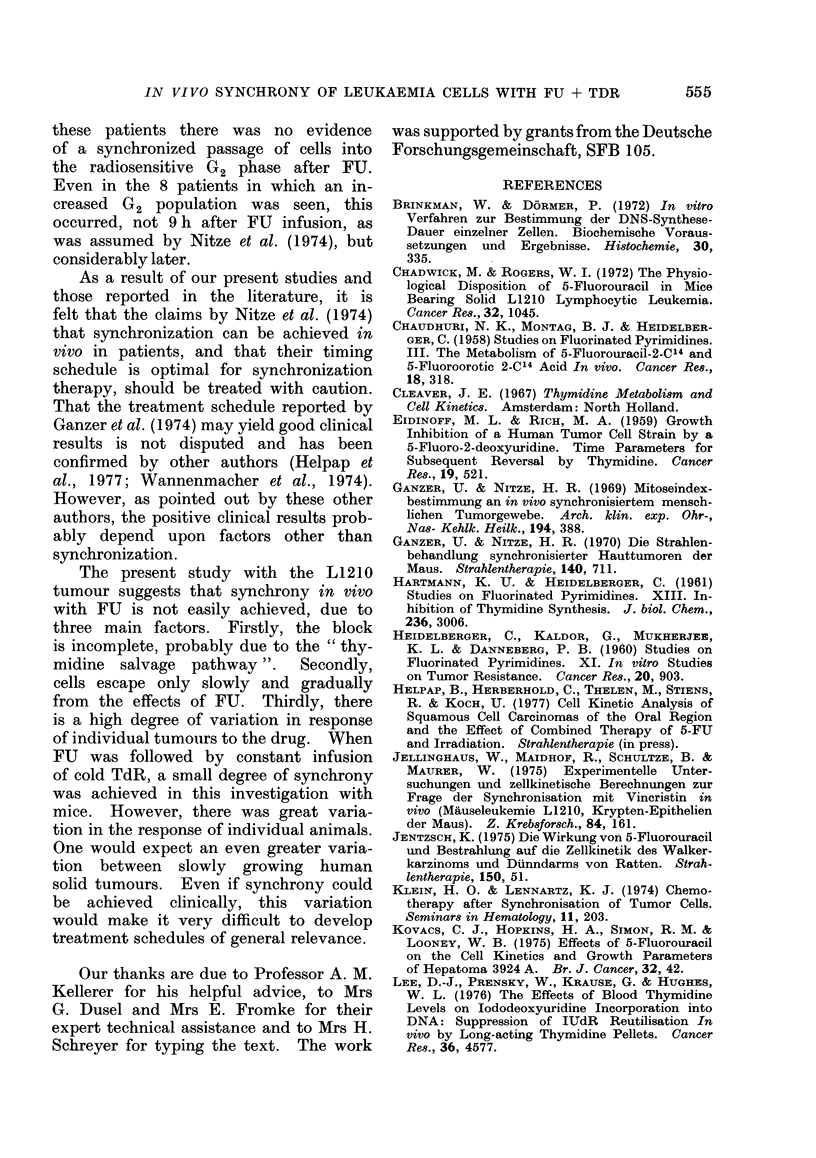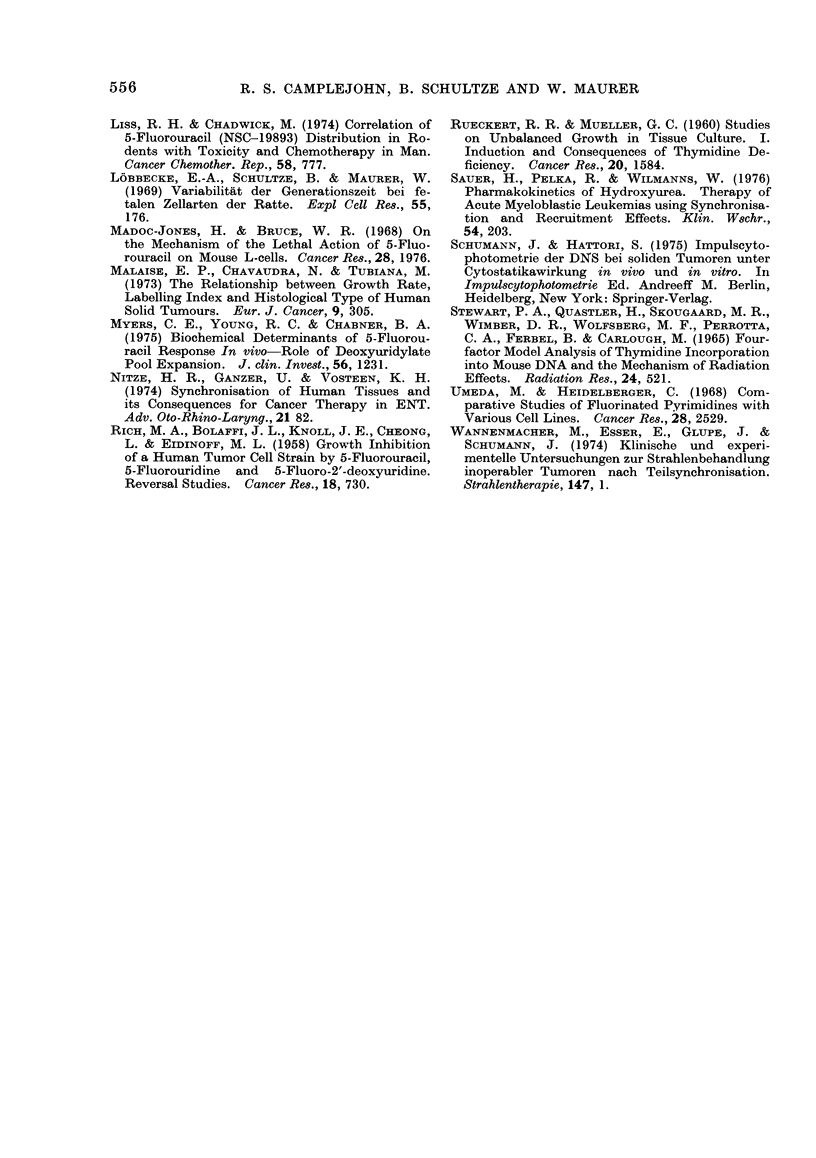# In vivo cell synchrony in the L1210 mouse leukaemia studied with 5-fluorouracil or 5-fluorouracil followed by cold thymidine infusion.

**DOI:** 10.1038/bjc.1977.88

**Published:** 1977-05

**Authors:** R. S. Camplejohn, B. Schultze, W. Maurer

## Abstract

[3H]-TdR and [3]-udR labelling indices and mitotic indices were followed in tumour-bearing mice after application of either 5-fluorouracil (FU) alone or of FU followed by cold TdR infusion. With FU alone, accumulation of cells at the beginning of S was found, but there was no indication of a synchronous passage of the accumulated cells further round the cycle. When FU injection was followed by cold TdR infusion, a synchronous passage of the accumulated cells through the cycle was observed. However, there was a large variation in the response of individual mice to this treatment.


*Br. J. Cancer* (1977) **35**, 546

### *IN VIVO* CELL SYNCHRONY IN THE L1210 MOUSE LEUKAEMIA STUDIED WITH 5-FLUOROURACIL OR 5-FLUOROURACIL FOLLOWED BY COLD THYMIDINE INFUSION

R. S. CAMPLEJOHN, B. SCHULTZE AND W. MAURER

*From the Institut für Medizinische Strahlenkunde der Universität Würzburg*

Received 20 December 1976 Accepted 12 January 1977

**Summary.—[$^3$H]-TdR and [$^3$H]-UdR labelling indices and mitotic indices were followed in tumour-bearing mice after application of either 5-fluorouracil (FU) alone or of FU followed by cold TdR infusion. With FU alone, accumulation of cells at the beginning of S was found, but there was no indication of a synchronous passage of the accumulated cells further round the cycle. When FU injection was followed by cold TdR infusion, a synchronous passage of the accumulated cells through the cycle was observed. However, there was a large variation in the response of individual mice to this treatment.**

In recent years, the concept of cell-cycle-specific therapy of tumours following synchronization of the tumour cells has found increasing attention. Three substances which have been used clinically to achieve synchrony are hydroxyurea (Sauer, Pelka and Wilmanns, 1976), vincristine (Klein and Lennartz, 1974) and 5-fluorouracil (FU) (Nitze, Ganzer and Vosteen, 1974). However, the efficacy of vincristine as a synchronizing agent has been disputed (Jellinghaus *et al.*, 1975). In addition, Wannenmacher *et al.* (1974) were unable to confirm the results obtained by Nitze *et al.* (1974) with FU.

As this form of clinical synchronization therapy is being widely used, it was decided to carry out the present study with FU. The aim of the present study was to see if *in vivo* synchrony can, in fact, be achieved with FU. For this purpose, the mouse L1210 leukaemia was used and the study was carried out in two parts. In the first part the effect of FU alone on the tumour cells was studied. In the second part FU injection was followed by a constant infusion of cold TdR. This was done to see whether the degree of synchrony could be improved by cold TdR infusion. The possibility of improving synchrony with cold TdR *in vitro* has already been demonstrated (Eidinoff and Rich, 1959; Rueckert and Mueller, 1960).

In a strict sense one can speak about synchronization only if two important conditions are fulfilled. The first condition is that an efficient accumulation of cells at a particular point in the cell cycle be produced. As is described later, the action of FU meets this condition, at least in part. However, it will also be seen that the action of FU alone does not meet the second condition, that after a suitable time a rapid dissolution of the block occurs, leading to a sharp release of accumulated cells and their subsequent synchronous passage round the cycle.

### MATERIAL AND METHODS

5-Fluorouracil (FU) was obtained from Hoffmann–La Roche, Grenzach/Baden.

[$^3$H-Methyl] thymidine ([$^3$H]-TdR, sp. act. 6·7 Ci/mmol) and [$^3$H-6]-deoxyuridine [$^3$H]-UdR, sp. act. 25·9 Ci/mmol) were obtained from New England Nuclear Chemicals, Boston, Mass., USA.

Cold TdR was purchased from the Sigma Chemical Company, St Louis, USA.

*Animals.*—The experiments were carried out on female B6D2 F1 mice (Laboratory of Animal Breeding and Research Centre, Gl Bomholtgard Ltd, Ry, Denmark). The mice weighed 20–27 g. They were kept under constant conditions (12-h light–dark regime, Altromin R standard food, water *ad lib.*).

*Transplantation of L1210 ascites tumour.*—Initially the tumour was transplanted at 6-day intervals. Ascitic fluid was pooled from 3 mice. Each recipient mouse received $10^5$ cells i.p. in a volume of 0·1 ml. Experiments were begun on the 5th day after transplantation.

The above method was used for the part of the study which will be described first. After 180 passages the L1210 cell line maintained in this institute ceased to grow normally, and new cells had to be obtained from Dr Bierling, Inst. f. Onkologie und Immunologie, Hauptfabriken Bayer, Wuppertal-Elberfeld, Germany. For the second part of the study (cold TdR infusion experiments), the transplantation procedure carried out at Bayer was adopted. The tumour was transplanted at weekly intervals and each recipient mouse received $2 \times 10^5$ cells in a volume of 0·2 ml. Experiments were begun on the 6th day after transplantation. The mean survival time of mice transplanted in this way is 12·5 days. During the experimental period, the mitotic index remained constant in untreated animals and there was only a small fall in labelling index.

*Constant infusion technique.*—The constant infusion technique was carried out as described by Löbbecke, Schultze and Maurer (1969). A catheter was inserted into the tail vein of mice on the day before the experiment began. For 24 h before the start of the experiment physiological saline was infused. At the appropriate time, the infusion medium for mice to receive cold TdR was changed to TdR solution (36 mg TdR in 20 ml 5% glucose solution). The control mice (without TdR) were maintained for the duration of the experiment on the physiological saline infusion. All infusions were given at a rate of 2·4 ml/day. During the experiment the mice were able to move around freely, and eat and drink at will.

To obtain samples of ascites fluid from the mice under constant infusion, a small-bore needle (Nr. 18 or 20) was carefully inserted into the peritoneal cavity and one drop of ascites fluid withdrawn. This procedure was repeated 8–10 times during the course of the experiment.

*Histology and autoradiography.*—Smears were prepared from the ascites, air-dried, fixed in methanol and Feulgen-stained. Autoradiographs were prepared by the dipping technique, with Ilford K2 emulsion. They were exposed at 4°C and developed with Amidol.

*Evaluation of slides.*—Mitotic and labelling indices were determined by counting a minimum of 1000 cells for each animal or, in the case of multiple-sampled mice, 1000 cells for each individual sample.

*Biochemical action of 5-fluorouracil*

The main effect of FU is to block the enzyme TdR synthetase (TS), which by methylation converts deoxyuridine phosphate to deoxythymidine phosphate (see Fig. 1). When deprived of deoxythymidine phosphate, cells are unable to synthesize DNA and therefore accumulate at the beginning of S. The active metabolite is not, however, FU itself but FUdRP. FU can also affect RNA synthesis, but the doses required to produce an observable effect on RNA synthesis are considerably higher (Heidelberger *et al.*, 1960; Hartmann and Heidelberger, 1961).

An important factor limiting the efficiency of accumulation of cells by FU is the so-called “ thymidine salvage pathway ”. Through this pathway cells are able to take up from their surroundings and reutilize TdR produced by catabolism of dead cells. By the action of TdR kinase (TK), the TdR enters the normal pathway of DNA synthesis as deoxythymidine phosphate (Fig. 1). It is well known that, particularly *in vivo*, this pathway can provide proliferating cells with a significant proportion of their TdR requirements. (See for example Cleaver, 1967).

As can be seen in Fig. 1, TdR enters the DNA synthesis pathway at a point after the action of TS, and its incorporation is thus unaffected by FU. This fact also explains an important difference between the incorporation of [$^3$H]-UdR and [$^3$H]-TdR into cells treated with FU. [$^3$H]-TdR incorporation does not proceed *via* the enzyme TS, and thus no reduction of incorporation is to be expected after application of FU.

FIG. 1.—Metabolism of 5-fluorouracil.

The block of this enzyme cannot therefore be directly measured with [$^3$H]-TdR. In contrast, [$^3$H]-UdR incorporation can only proceed *via* TS (Fig. 1) and this compound does allow a direct assessment of the efficiency of the FU block.

After a period of FU-induced blockage of DNA synthesis it should be possible to release cells more quickly from the effects of the block by supplying them with exogenous TdR. If sufficient TdR is available, it can be incorporated by the action of TK into the main DNA synthesis pathway and, even with a fully blocked *de novo* synthesis of deoxythymidine phosphate, allow cells to progress through the S phase. By this means, a sharper release of cells from the FU block should be achieved. As stated earlier, this has indeed been demonstrated *in vitro* (Eidinoff and Rich, 1959; Rueckert and Mueller, 1960).

### RESULTS

### *Determination of FU dose*

The first part of this investigation involved experiments to determine a suitable dose of FU which results in an effective but reversible block of cells at the beginning of S. To assess the response of the L1210 tumour cells to a range of FU doses (0·1; 1; 3; 10; 30; 100 $\mu$g/g body wt) mitotic and [$^3$H]-UdR-labelling indices were determined before, and at various times (1; 10; 48 h) after, injection of FU.

The results for doses of 0·1, 3, 30 and 100 $\mu$g/g FU are illustrated in Fig. 2. A dose of 0·1 $\mu$g/g caused a sharp initial fall in mitotic index (Fig. 2(*a*)). In

FIG. 2.—Response of L1210 tumour cells to a range of FU doses (0·1; 3; 30 and 100 $\mu$g/g). (a) Mitotic index, (b) [$^3$H]-UdR-labelling index as a function of time after FU injection. For simplicity, experimental points are not shown. For each dose, indices were measured before and at 1·5, 10 and 48 h after FU injection. The number of mice per time point varied from 2 to 9 for all doses studied. The full data for 3 $\mu$g/g FU is given in Fig. 3.

addition, [$^3$H]-UdR grain count wa reduced to about half of the norma value 1·5 h after application of FU However, there was no depression o [$^3$H]-UdR-labelling index following FU (Fig. 2(*b*)). The block of TS was clearl far from complete. Doses of 100 an 30 $\mu$g/g FU caused an irreversible decreas of both mitotic and [$^3$H]-UdR-labellin indices. No recovery of the proliferativ indices was seen for the duration of th experiment (48 h). Moreover, there wa evidence of cell killing with these tw highest doses. In contrast, a dose o 3 $\mu$g/g FU caused an initial sharp de pression of both mitotic and labellin indices, followed by a peak of 80% labelled cells 10 h after FU, and norma indices 48 h after FU. As with th other doses in the range 0·1 to 10 μg/g, no evidence of cell killing was seen.

One μg/g and 10 μg/g FU had an effect on the mitotic index similar to that of 3 μg/g. Differences resulted, however, in the response of the [$^3$H]-UdR-labelling index. Though there was a marked reduction in grain count per nucleus 1·5 h after a dose of 1 μg/g, the labelling index was little affected; thus the block of TS was less effective than with a dose of 3 μg/g. After application of 10 μg/g FU, recovery of labelling index was much slower than after a dose of 3 μg/g. Ten hours after FU injection, only 10% [$^3$H]-UdR-labelled cells were found (in contrast to nearly 80% labelled cells with 3 μg/g FU).

On the basis of these results, 3 μg/g FU was chosen as the most likely dose to give a synchronizing effect, as it caused a substantial initial block followed later by recovery of proliferative indices. This dose lies between the highest doses, which caused an irreversible block and resulted in cell killing, and the lower doses, which were less effective in blocking TS.

*Action of* 3 *μg/g FU alone*

After the choice of 3 μg/g FU as the most suitable dose, more extensive studies of the mitotic index and the incorporation of [$^3$H]-UdR and [$^3$H]-TdR were made. Fig. 3 shows the results.

(*a*) *Mitotic index.*—Fig. 3(a) demonstrates the mitotic index for singly sampled mice; groups of mice were killed at the various time points and a single sample of ascites fluid withdrawn. As can be seen, the curve shows a sharp decrease, reaching a minimum 8 h after injection of FU. This trough is followed by a slow rise to approximately normal values, with no evidence of a mitotic peak.

To see whether repeated withdrawal of ascites fluid affected the mitotic index *per se*, an experiment was carried out in which 4 mice were given 3 μg/g FU, and each mouse was then sampled 10

FIG. 3.—The effect of 3 μg/g FU on (a) mitotic index; (b) [$^3$H]-UdR-labelling index, (c) [$^3$H]-TdR labelling index. The dashed line in (c) shows the [$^3$H]-TdR-labelling index over this period in the absence of 5-FU. Each point represents the mean value of 2–9 animals; where 3 or more animals were present in a group, s.e. mean is shown.

FIG. 4.—Mitotic index with multiple sampling following 3 μg/g FU. The solid line shows the course of the mitotic index in mice sampled only once. This curve is given fully, with individual points and s.e. in Fig. 3(a). The points in this figure are the mean values for a group of 4 mice, from each of which 10 small samples of ascites were withdrawn during the experimental period; s.e. mean indicated.

times over a period of 35 h. The results are given in Fig. 4. The course of the mitotic index for multiply-sampled mice (points, Fig. 4) was found to be essentially the same as obtained from groups of singly-sampled mice (curve, Fig. 4). Thus, multiple sampling as carried out here had no significant effect on the mitotic index.

(*b*) [$^3$*H*]-*UdR labelling*.—The [$^3$H]-UdR-labelling index curve is given in Fig. 3(b). As described previously, a sharp fall is seen after injection of FU, and this is followed by a peak of almost 80% labelled cells after 10 h, and nearly normal values after 48 hours. A minimum of 10% labelled cells is seen at 5 h; the few labelled cells at this time have a median grain count only one-tenth the value of controls without FU, and thus [$^3$H]-UdR incorporation is more sharply reduced than the labelling index alone would indicate. Recovery of grain count to normal values is slow: although 80% labelled cells are seen 10 h after FU, the median grain count is only one 6th of control values. At 48 h the median grain count in FU-treated mice is about three-quarters the control level.

(*c*) [$^3$*H*]-*TdR*.—The [$^3$H]-TdR-labelling results after 3 $\mu$g/g FU are illustrated in Fig. 3(c). As expected, no fall in [$^3$H]-TdR-labelling index was seen after injection of FU: instead there was a steady rise to a peak value of about 80% at 8 h. This value was similar to that obtained with [$^3$H]-UdR at the same time. The grain count remained approximately normal over the period of the experiment. After the peak labelling index, a slow decrease occurred, normal values being reached about 20 h after FU injection.

*FU application followed by cold TdR infusion*

As was stated earlier, it is possible to achieve *in vitro* partial synchrony, which persists at least up to the subsequent mitosis, by sharpening the release of cells from the FU block with thymidine.

FIG. 5.—Cold TdR constant infusion experiments. Experimental design and mitotic curves for a group of 8 mice (with TdR (solid line) and 6 mice without TdR (dotted line). s.e. indicated.

To examine this possibility *in vivo*, FU injection in tumour-bearing mice was followed by constant infusion of cold TdR.

(*a*) *Experimental design and results*.—Fig. 5 illustrates the experimental design and also the mean mitotic index curves obtained for a group of 8 experimental and 6 control mice. All mice were given 3 $\mu$g/g FU i.p. at the beginning of the experiment and again 2·5 h later. The second injection was given to ensure a maximum block for the 5-h period calculated to be necessary for entry of all $G_1$, $G_2$ and M cells into S. Continuous infusion of FU over this 5-h period was tried but found not to improve accumulation of cells at the beginning of S.

Five hours after the first FU injection, one group of 8 mice was infused i.v. with cold TdR, while a control group of 6 mice was maintained on physiological saline infusion. The rate of TdR infusion was 3 $\mu$g/mouse/min. This rate was calculated, from the work of Stewart *et al.* (1965) and Lee *et al.* (1976) to be sufficient to provide the mice with enough TdR for normal requirements in the absence of *de novo* synthesis. A drop of ascitic fluid was withdrawn from each mouse 8 times during a 28-h period. The mitotic index was determined for all samples.

The solid line (Fig. 5) indicates the course of the mitotic index for the mice which received FU followed by cold TdR: the points are mean values for the group of 8 mice; standard errors are shown. For comparison, the dotted line gives the resulting means for the group of 6 control mice given FU but no cold TdR. As can be seen, a peak mitotic index was obtained in the cold TdR-treated mice. In fact, the cold TdR group have a significantly higher mitotic index over the period 8 to 23 h after the first FU injection (Mann–Whitney test). The peak mitotic index in the cold TdR group occurred at 19 h

FIG. 6.—Mitotic index curves for individual cold TdR mice. These mice received FU followed by cold TdR infusion. Each solid line represents one experimental mouse. The broken line in each graph is the mean for 6 mice without TdR.

after the first FU injection. This is 14 h after the beginning of the cold TdR infusion. If cells blocked at the beginning of S had passed at the normal rate around the cycle at the start of cold TdR infusion, they would have been expected to reach mitosis in a time equal to $S + G_2 + \frac{1}{2}M$; this is about 8–9 h in this tumour. Thus, the peak of mitoses occurred somewhat later than expected.

(*b*) *Mitotic results for individual mice infused with cold TdR.*—The variation in response of individual mice to FU treatment followed by cold TdR infusion is seen in Fig. 6. The mitotic curves for the 8 cold TdR mice are given by the solid lines. These are the individual curves, whose average is plotted as the solid line in Fig. 5. Once again, for comparison, the mean curve for the control group is given in each case. As can be seen, not only does the height of the mitotic peak vary greatly, but also its timing and shape.

(*c*) *Labelling index results.*—In addition to the mitotic index measurements, [$^3$H]-TdR-labelling indices were also determined in both the control and cold TdR groups of mice. It was, of course, only possible to measure the [$^3$H]-TdR labelling index at one time for each mouse. The labelling index was measured in 4 mice, 5 h after the first FU injection. This was done to assess the percentage of cells accumulated at the beginning of S in this 5-h period. At the time of the mitotic peak in the cold TdR mice (Fig. 5), 19 h after the first FU injection, the labelling index was also determined for groups of 4 control and 4 cold-TdR mice.

At the end of the FU block period (Table; 5 h) approximately 70% labelled cells were seen. This is about 20% more than the normal value of 50% (Table; 0 h) and indicates an accumulation of 20% of the cells at the beginning of S. Nineteen hours after the first FU injection the control mice without cold TdR had a mean mitotic index of 1·8%, and no

TABLE.—[$^3H$]-*TdR-labelling Indices Following FU Application Alone and FU Application Plus Cold TdR Infusion*

| Animal group | Number of mice | h after FU injection | Labelling index % (mean±s.e.) |
|---|---|---|---|
| Untreated control | 6 | 0 | 50·0±2·5 |
| FU only | 4 | 5 | 68·0±2·6 |
| FU only | 4 | 19 | 70·0±3·9 |
| FU+cold TdR | 4 | 19 | 49·1±3·6 |

evidence of a mitotic peak (Fig. 5—dotted line). At this time, the labelling index was still 70%, indicating that the accumulated cells had not yet left the S phase. In contrast, the cold TdR mice 19 h after the first FU injection had a mean mitotic index of 4·4% (Fig. 5—solid line). At this time the mean labelling index for the cold TdR mice was reduced to 49% (Table; 19 h). This is further evidence that the cold TdR infusion had, indeed, led to a faster release of the cells blocked by FU. The accelerated passage of these cells through the S phase into mitosis leads to the mitotic peak shown in Fig. 5 (solid line).

### DISCUSSION

### *Action of FU alone*

The mitotic and labelling results (Fig. 3(a–c)) with FU alone, showed that we achieved a considerable, but not complete, block of cells at the beginning of the S phase. If a complete block had been achieved, the mitotic index would have reached zero. It is known that the rate of entry of cells into S in this tumour is 7%/h: thus, after 5 h one would have expected to have accumulated 35% of the cells at the beginning of S, if the block had been complete. The [$^3$H]-TdR curve (Fig. 3(c)) shows that, in fact, a little over 20% of the cells were accumulated. The [$^3$H]-UdR-labelling results (Fig. 3(b)) indicated that over the first 5 h after FU the block of TS was almost complete (only 10% very lightly labelled cells were seen at 5 h). The main factor preventing a better block is almost certainly the TdR available to the cells *via* the " thymidine salvage " pathway.

The [$^3$H]-UdR and mitotic index curves (Fig. 3(a) and (b)) suggest that cells begin to escape from the block by about 10 h after application of FU, but this release is slow and gradual. There is no evidence (Fig. 3(c)) of a second peak of labelled cells. Furthermore, no peak of mitotic index is seen after the labelling-index peak. Instead, the mitotic index rises slowly, reaching normal values 30 h after FU injection. If accumulated cells had been released sharply, they would have required a period equal to the duration of S and $G_2$ to reach mitosis: about 8–9 h in the L1210 tumour. Under this condition, a peak of mitotic activity would have been seen 16 to 20 h after FU. This was clearly not the case (Fig. 3(a)). Thus, the cells accumulated at the beginning of S do not pass through mitosis in a synchronized wave, and it would seem that FU does not, at least in this study, fulfil the second requirement of a synchronizing agent (*i.e.* the accumulated cells do not escape sharply from the block and do not pass synchronously further round the cycle).

This failure of the accumulated cells to pass synchronously round the cycle could be due either to a slow dissolution of the block or to their sustaining permanent damage leading to a complete failure to proliferate further, and eventual death. With a dose of 3 $\mu$g/g FU, no cytological evidence of cell death was seen, in contrast to higher doses. Thus, the most likely explanation for the failure to achieve synchrony was a slow release of cells from the block. Schumann and Hattori (1975) found, with a hamster sarcoma cell line *in vitro*, that passage of cells round the cycle after FU treatment was markedly slowed. They found that cells which normally had an S phase of 8–10 h required 72 h to pass from the beginning of S to $G_2$ after FU.

A possible reason for the slow recovery of cells from the effects of FU may be the long life of active FU metabolites, particularly FUdRP, in the tissues. Although a large part of injected FU is excreted rapidly (Chaudhuri, Montag and Heidelberger, 1958), evidence from studies with $^{14}$C-labelled FU suggests that active metabolites are present in the tissues for periods up to 72 h after injection (Chadwick and Rogers, 1972; Liss and Chadwick, 1974). FU has also been shown to have long-lasting effect on [$^3$H]-UdR incorporation into tumour cells (Kovacs *et al.*, 1975; Myers, Young and Chabner, 1975). Myers *et al.* (1975) found that a dose of 15 $\mu$g/g FU caused depression of [$^3$H]-UdR incorporation in an ascites tumour, lasting 72 h. These authors showed that the recovery of [$^3$H]-UdR incorporation depends not only on the rate of recovery of DNA synthesis but also on changes in metabolite pool sizes within the cells. Such biochemical factors may well explain the observed slow recovery of [$^3$H]-UdR grain count in the present study. If this is the case, [$^3$H]-UdR incorporation after FU application is not directly proportional to the rate of DNA synthesis. Thus, although [$^3$H]-UdR is useful to demonstrate onset of the block of DNA synthesis, because of changes in incorporation rate due to changes in metabolite pool sizes, [$^3$H]-UdR does not allow a direct assessment of the rate of recovery of DNA synthesis after FU.

*Effect of FU followed by cold TdR*

When injection of FU was followed by constant infusion with cold TdR, evidence of a synchronized passage of cells round the cycle was seen. In all animals which received cold TdR, there was an increase in mitotic index in the period 3–18 h after the beginning of the TdR infusion. The mitotic peaks were, however, generally broad and fairly small (Fig. 6). That the peaks were not higher is not surprising, in view of the fact that accumulation of cells by FU was only partial. Also, in view of the known variation of S phase duration in the L1210 tumour, considerable flattening of the mitotic peaks would be expected. The peak mitotic index in Fig. 5 occurred 14 h after the start of the cold TdR infusion. If the accumulated cells had passed through S and $G_2$ at the normal rate, they would have been expected to reach mitosis 8–9 h later (S + $G_2$). No firm explanation for this delay of about 5 h is available. One possibility is that the dose of cold TdR was not quite optimal, and therefore DNA synthesis took longer than normal. It is possible that, if a slightly higher dose had been given, the peak of mitoses would have been seen earlier. A range of cold TdR doses was not tested, simply due to the practical limitations of the constant infusion technique, which allows only a small number of mice to be infused at any one time.

In vitro *and* in vivo *studies with animals*

It has clearly been shown *in vitro* that the growth-blocking effect of FU or FUdR on proliferating cultured cells can be prevented by supplying the cells with exogenous TdR (Brinkmann and Dörmer, 1972; Madoc-Jones and Bruce, 1968; Rich *et al.*, 1958; Umeda and Heidelberger, 1968). Further, cells accumulated at the beginning of the S phase by these compounds have been shown, when released from this block by added TdR, to proceed in a partially synchronized wave round the cell cycle, at least as far as the subsequent mitosis (Eidinoff and Rich, 1959; Rueckert and Mueller, 1960).

*In vivo* studies in animals of the problem of synchronization with FU are few in number. Jentzsch (1975), using a dose of 13 $\mu$g/g FU with the rat Walker tumour, found no effect on mitotic or labelling index. An effect on the PLM* curve in animals given FU compared with controls was found, but is difficult to explain. Ganzer and Nitze (1970) and Nitze *et al* (1974) reported investigations on chemically induced skin tumours of

* % labelled metaphases.

mice. In these studies, life-span of the animals and rate of growth of the tumours were assessed for 5 groups of mice.

One group (" combination therapy " group) received FU and irradiation separated by an interval of one day, while another group (" synchronously " treated group) received FU over a 12-h period followed 8 h later by irradiation. It was assumed that the cells accumulated by FU escaped sharply from the block and passed in 8 h through S into the radiosensitive $G_2$ phase. It was found that the second, so-called " synchronously treated ", group had a longer survival time and that the tumours in this group grew more slowly than in the " combination therapy " group. These differences were ascribed to the effect of FU-induced synchronization. However, no direct evidence that synchrony had, in fact, been achieved was presented (*e.g.* no labelling or mitotic studies). There is no doubt that there are timing factors other than those relating to synchrony, which are important for the success of combination therapy. Thus caution should be exercized in ascribing effects such as those described above to synchrony.

*Clinical studies with FU*

FU has been used in the treatment of malignant tumours for many years, and there is no doubt that with certain types of tumour its use, either alone or in combination, can be clinically valuable. The literature on the clinical use of FU, particularly as part of combination therapy schedules, is extensive. Its clinical use as a synchronizing agent is, however, more recent. Extensive investigations in this area have been made by Nitze and co-workers (Ganzer and Nitze, 1969; Nitze *et al.*, 1974). The results of these studies are reviewed in the paper from Nitze *et al.* (1974). In these studies FU was infused into tumour-bearing patients for a period of 12 or 18 h and mitotic indices and labelling indices were determined by *in vitro* incubation with [$^3$H]-TdR at various times after cessation of the FU infusion. A rise in labelling index was found immediately after FU infusion, followed 8 h later by a fall. The increase was taken as due to accumulation of cells at the beginning of S, and the decline as showing the progress of these cells into the $G_2$ phase. Caution is perhaps necessary in interpretation of these labelling results, as the labelling was carried out *in vitro*, and some of the samples were incubated *in vitro* for up to 8 h before labelling. The longer the time *in vitro* the less directly can the values be assumed to relate to the *in vivo* value. The mitotic index curves obtained by these authors were interpreted as indicating synchrony; however, a number of anomalies are evident. Immediately at the end of the FU block, only a very slight fall in mitotic index was seen. If a sufficient block had been achieved to cause clear synchrony, one would have expected a more dramatic fall in mitotic index. The main evidence that synchrony had been achieved was a small mitotic peak 9 h after the end of the FU block. This peak was, however, small and its statistical significance doubtful. The timing of the peak is also of interest: the authors assumed an S-phase duration of 8 h, and they also assumed that the accumulated cells immediately after FU infusion began their passage round the cycle at the normal rate. From a survey of the literature, the assumption concerning the duration of S would not seem too secure. In human tumours this has generally been shown to be considerably longer than 8 h (see for example Malaise, Chavaudra and Tubiana, 1973). The assumption that the cells at the end of the block immediately begin to cycle at the normal rate is, from the results presented in this paper, equally doubtful.

Wannenmacher *et al.* (1974) treated 23 patients with squamous cell carcinoma with the therapeutic scheme of Nitze *et al.* (1974). Impulse cytophotometry was used to follow the kinetic changes in the tumours after FU infusion. In 15 of these patients there was no evidence of a synchronized passage of cells into the radiosensitive $G_2$ phase after FU. Even in the 8 patients in which an increased $G_2$ population was seen, this occurred, not 9 h after FU infusion, as was assumed by Nitze *et al.* (1974), but considerably later.

As a result of our present studies and those reported in the literature, it is felt that the claims by Nitze *et al.* (1974) that synchronization can be achieved *in vivo* in patients, and that their timing schedule is optimal for synchronization therapy, should be treated with caution. That the treatment schedule reported by Ganzer *et al.* (1974) may yield good clinical results is not disputed and has been confirmed by other authors (Helpap *et al.*, 1977; Wannenmacher *et al.*, 1974). However, as pointed out by these other authors, the positive clinical results probably depend upon factors other than synchronization.

The present study with the L1210 tumour suggests that synchrony *in vivo* with FU is not easily achieved, due to three main factors. Firstly, the block is incomplete, probably due to the " thymidine salvage pathway ". Secondly, cells escape only slowly and gradually from the effects of FU. Thirdly, there is a high degree of variation in response of individual tumours to the drug. When FU was followed by constant infusion of cold TdR, a small degree of synchrony was achieved in this investigation with mice. However, there was great variation in the response of individual animals. One would expect an even greater variation between slowly growing human solid tumours. Even if synchrony could be achieved clinically, this variation would make it very difficult to develop treatment schedules of general relevance.

Our thanks are due to Professor A. M. Kellerer for his helpful advice, to Mrs G. Dusel and Mrs E. Fromke for their expert technical assistance and to Mrs H. Schreyer for typing the text. The work was supported by grants from the Deutsche Forschungsgemeinschaft, SFB 105.

REFERENCES

BRINKMAN, W. & DÖRMER, P. (1972) *In vitro* Verfahren zur Bestimmung der DNS-Synthese-Dauer einzelner Zellen. Biochemische Voraussetzungen und Ergebnisse. *Histochemie*, **30**, 335.

CHADWICK, M. & ROGERS, W. I. (1972) The Physiological Disposition of 5-Fluorouracil in Mice Bearing Solid L1210 Lymphocytic Leukemia. *Cancer Res.*, **32**, 1045.

CHAUDHURI, N. K., MONTAG, B. J. & HEIDELBERGER, C. (1958) Studies on Fluorinated Pyrimidines. III. The Metabolism of 5-Fluorouracil-2-$C^{14}$ and 5-Fluoroorotic 2-$C^{14}$ Acid *In vivo*. *Cancer Res.*, **18**, 318.

CLEAVER, J. E. (1967) *Thymidine Metabolism and Cell Kinetics.* Amsterdam: North Holland.

EIDINOFF, M. L. & RICH, M. A. (1959) Growth Inhibition of a Human Tumor Cell Strain by a 5-Fluoro-2-deoxyuridine. Time Parameters for Subsequent Reversal by Thymidine. *Cancer Res.*, **19**, 521.

GANZER, U. & NITZE, H. R. (1969) Mitoseindexbestimmung an *in vivo* synchronisiertem menschlichen Tumorgewebe. *Arch. klin. exp. Ohr-, Nas- Kehlk. Heilk.*, **194**, 388.

GANZER, U. & NITZE, H. R. (1970) Die Strahlenbehandlung synchronisierter Hauttumoren der Maus. *Strahlentherapie*, **140**, 711.

HARTMANN, K. U. & HEIDELBERGER, C. (1961) Studies on Fluorinated Pyrimidines. XIII. Inhibition of Thymidine Synthesis. *J. biol. Chem.*, **236**, 3006.

HEIDELBERGER, C., KALDOR, G., MUKHERJEE, K. L. & DANNEBERG, P. B. (1960) Studies on Fluorinated Pyrimidines. XI. *In vitro* Studies on Tumor Resistance. *Cancer Res.*, **20**, 903.

HELPAP, B., HERBERHOLD, C., THELEN, M., STIENS, R. & KOCH, U. (1977) Cell Kinetic Analysis of Squamous Cell Carcinomas of the Oral Region and the Effect of Combined Therapy of 5-FU and Irradiation. *Strahlentherapie* (in press).

JELLINGHAUS, W., MAIDHOF, R., SCHULTZE, B. & MAURER, W. (1975) Experimentelle Untersuchungen und zellkinetische Berechnungen zur Frage der Synchronisation mit Vincristin *in vivo* (Mäuseleukemie L1210, Krypten-Epithelien der Maus). *Z. Krebsforsch.*, **84**, 161.

JENTZSCH, K. (1975) Die Wirkung von 5-Fluorouracil und Bestrahlung auf die Zellkinetik des Walkerkarzinoms und Dünndarms von Ratten. *Strahlentherapie*, **150**, 51.

KLEIN, H. O. & LENNARTZ, K. J. (1974) Chemotherapy after Synchronisation of Tumor Cells. *Seminars in Hematology*, **11**, 203.

KOVACS, C. J., HOPKINS, H. A., SIMON, R. M. & LOONEY, W. B. (1975) Effects of 5-Fluorouracil on the Cell Kinetics and Growth Parameters of Hepatoma 3924 A. *Br. J. Cancer*, **32**, 42.

LEE, D.-J., PRENSKY, W., KRAUSE, G. & HUGHES, W. L. (1976) The Effects of Blood Thymidine Levels on Iododeoxyuridine Incorporation into DNA: Suppression of IUdR Reutilisation *In vivo* by Long-acting Thymidine Pellets. *Cancer Res.*, **36**, 4577.

LISS, R. H. & CHADWICK, M. (1974) Correlation of 5-Fluorouracil (NSC–19893) Distribution in Rodents with Toxicity and Chemotherapy in Man. *Cancer Chemother. Rep.*, **58**, 777.

LÖBBECKE, E.-A., SCHULTZE, B. & MAURER, W. (1969) Variabilität der Generationszeit bei fetalen Zellarten der Ratte. *Expl Cell Res.*, **55**, 176.

MADOC-JONES, H. & BRUCE, W. R. (1968) On the Mechanism of the Lethal Action of 5-Fluorouracil on Mouse L-cells. *Cancer Res.*, **28**, 1976.

MALAISE, E. P., CHAVAUDRA, N. & TUBIANA, M. (1973) The Relationship between Growth Rate, Labelling Index and Histological Type of Human Solid Tumours. *Eur. J. Cancer*, **9**, 305.

MYERS, C. E., YOUNG, R. C. & CHABNER, B. A. (1975) Biochemical Determinants of 5-Fluorouracil Response *In vivo*—Role of Deoxyuridylate Pool Expansion. *J. clin. Invest.*, **56**, 1231.

NITZE, H. R., GANZER, U. & VOSTEEN, K. H. (1974) Synchronisation of Human Tissues and its Consequences for Cancer Therapy in ENT. *Adv. Oto-Rhino-Laryng.*, **21** 82.

RICH, M. A., BOLAFFI, J. L., KNOLL, J. E., CHEONG, L. & EIDINOFF, M. L. (1958) Growth Inhibition of a Human Tumor Cell Strain by 5-Fluorouracil, 5-Fluorouridine and 5-Fluoro-2′-deoxyuridine. Reversal Studies. *Cancer Res.*, **18**, 730.

RUECKERT, R. R. & MUELLER, G. C. (1960) Studies on Unbalanced Growth in Tissue Culture. I. Induction and Consequences of Thymidine Deficiency. *Cancer Res.*, **20**, 1584.

SAUER, H., PELKA, R. & WILMANNS, W. (1976) Pharmakokinetics of Hydroxyurea. Therapy of Acute Myeloblastic Leukemias using Synchronisation and Recruitment Effects. *Klin. Wschr.*, **54**, 203.

SCHUMANN, J. & HATTORI, S. (1975) Impulscytophotometrie der DNS bei soliden Tumoren unter Cytostatikawirkung *in vivo* und *in vitro*. In *Impulscytophotometrie* Ed. Andreeff M. Berlin, Heidelberg, New York: Springer-Verlag.

STEWART, P. A., QUASTLER, H., SKOUGAARD, M. R., WIMBER, D. R., WOLFSBERG, M. F., PERROTTA, C. A., FERBEL, B. & CARLOUGH, M. (1965) Four-factor Model Analysis of Thymidine Incorporation into Mouse DNA and the Mechanism of Radiation Effects. *Radiation Res.*, **24**, 521.

UMEDA, M. & HEIDELBERGER, C. (1968) Comparative Studies of Fluorinated Pyrimidines with Various Cell Lines. *Cancer Res.*, **28**, 2529.

WANNENMACHER, M., ESSER, E., GLUPE, J. & SCHUMANN, J. (1974) Klinische und experimentelle Untersuchungen zur Strahlenbehandlung inoperabler Tumoren nach Teilsynchronisation. *Strahlentherapie*, **147**, 1.